# Assessment of Sensory Processing and Executive Functions in Childhood: Development, Reliability, and Validity of the EPYFEI

**DOI:** 10.3389/fped.2018.00071

**Published:** 2018-03-23

**Authors:** Dulce Romero-Ayuso, Sara Jorquera-Cabrera, Antonio Segura-Fragoso, Abel Toledano-González, M. Carmen Rodríguez-Martínez, José Matías Triviño-Juárez

**Affiliations:** ^1^Division of Occupational Therapy, Department of Physical Therapy, Faculty of Health Sciences, University of Granada, Granada, Spain; ^2^AYTONA Children’s Therapy Centre, Madrid, Spain; ^3^Department of Medical Science, Faculty of Occupational Therapy, Speech Therapy and Nursing, University of Castilla-La Mancha, Talavera de la Reina, Spain; ^4^Department of Psychology, Faculty of Occupational Therapy, Speech Therapy and Nursing, University of Castilla-La Mancha, Talavera de la Reina, Spain; ^5^Division of Occupational Therapy, Department of Physical Therapy, Faculty of Health Sciences, University of Malaga, Malaga, Spain; ^6^Primary Care Center Zaidín Sur, Andalusian Health Service, Granada, Spain

**Keywords:** sensory processing, executive functions, children, psychometrics, validity, reliability

## Abstract

The aim of this study was to determine the psychometric properties of the “Assessment of Sensory Processing and Executive Functions in Childhood” (EPYFEI), a questionnaire designed to assess the sensory processing and executive functions of children aged between 3 and 11 years. The EPYFEI was completed by a sample of 1,732 parents of children aged between 3 and 11 years who lived in Spain. An exploratory factor analysis was conducted and showed five main factors: (1) executive attention, working memory, and initiation of actions; (2) general sensory processing; (3) emotional and behavioral self-regulation; (4) supervision, correction of actions, and problem solving; and (5) inhibitory. The reliability of the analysis was high both for the whole questionnaire and for the factors it is composed of. Results provide evidence of the potential usefulness of the EPYFEI in clinical contexts for the early detection of neurodevelopmental disorders, in which there may be a deficit of executive functions and sensory processing.

Occupational therapy interventions and their evaluation in children have particularly focused on assessing sensory processing (1). Sensory processing is considered a key element to understand participation in various daily activities in relation to contextual demands (2, 3). The brain receives sensory information, builds a representation of the world around us, and gives us the ability to react and interact optimally with the environment (4). The prevalence of sensory processing disorders is estimated to range between 5 and 16% in the general population, reaching 90% in autism spectrum disorder (ASDs) (5–7). In addition to sensory processing, assessments should consider the cognitive factors that may affect the performance of individuals in various activities, including personal care, academic achievement, play, or social participation (1, 8). In this cognitive area it is particularly relevant to assess executive functions (1, 9). Although several definitions of executive functions exist, there is agreement on the need to consider several essential elements: (1) So-called “cold” executive functions, which include inhibitory control, attention span, working memory, and the planning, execution, and monitoring of actions (10, 11); and (2) “hot” executive functions, which include the self-regulation of behavior, the experience of reinforcement and punishment, and decision making based on the individual’s emotional and personal interpretation (11–14). Executive functions enable intentional and purposeful behavior, which is directed toward achieving a goal (15). It is, therefore, understood that a deficit of executive functions can have devastating effects on activities of daily life that could be interpreted as human occupations in a broad sense, such as school activities, self-care, play, leisure, and social participation (11, 16, 17). A typical development of executive functions is considered to be a protective factor in various neurodevelopmental disorders (18, 19). By contrast, children with executive function deficits are highly likely to be diagnosed with a neurodevelopmental disorder in the future (18, 19). It has been observed that the sensory symptoms of school-aged children with autism are affected by the degree of cognitive maturity of the children (9, 20). A strong and positive correlation has also been observed between difficulties in sensory processing and executive function deficits in various populations, such as premature children (21–23), children with ASDs (24–26), and children with attention deficit hyperactivity disorder (ADHD) (27–29). These results show that difficulties associated with sensory processing and executive functions tend to be related to each other. It has even been suggested that inhibitory control and executive attention play an important role in the regulation of sensory processing as a latent factor (19) and that tactile sensitivity is also a predictor of the self-regulation of behavior (30).

It would be interesting to take the interrelation between sensory processing and executive functions as a basis to develop standardized evaluation tools that could provide insight on the factors of both sensory processing and executive functions together. This would lead to a more holistic view of the causes of difficulties in learning, behavior, and functionality in activities of daily living in children (31–33). Such standard tests might allow us to obtain an individual profile of the strengths and weaknesses of children that could help us make comparisons with the normative data of the typical population (7, 33). Moreover, tests that obtain information from parents, teachers, or carers have a greater ecological validity than laboratory tests, since they focus on daily life, which is consistent with a family centered approach (33, 34).

To the best of the authors’ knowledge, to date there are no questionnaires available to jointly and simultaneously assess sensory processing and executive functions, considering the interrelationships between both processes in children’s daily activities. This type of instrument would make it easier to obtain an overall view of the influence that sensory processing and executive functions have on individuals’ functional performance in several areas. Examples of such areas are the quality and quantity of play, social participation (35), family routines, and everyday activities, particularly dressing and feeding (36, 37). The assessment of children’s performance in these areas would contribute to the planning of treatment.

Considering this, the main objective of this study was to develop a standardized instrument to assess sensory processing and executive functions in children aged 3–11 years; we expected such an instrument to enable early detection of neurodevelopmental disorders based on the information provided by caregivers on the participation of children in their various daily activities. This instrument, the “Assessment of Sensory Processing and Executive Functions in Childhood” (EPYFEI) was also intended to provide a response to the lack of validated instruments for the Spanish population. This new tool was called EPYFEI as an acronym of the full name of the instrument in Spanish: *Evaluación del Procesamiento sensorial Y Funcionamiento Ejecutivo en la Infancia*.

## Methods

The methods used to design and assess the metric properties of the EPYFEI questionnaire were based on the quality criteria for measuring properties in questionnaires about health status (38).

### Content Validity

The EPYFEI was developed by first conducting a literature review, followed by a meeting attended by five occupational therapists and one neuropsychologist in 2015. The first draft of the questionnaire included 108 items, which were created according to the various dimensions of sensory processing and executive functions. Two rounds of consultations then took place with three occupational therapists who were experts in sensory integration and children’s therapy. One of them was from the United States (University of Southern California) and the other two were Spanish professionals with more than 10 years experience in the field. The first round enabled the experts to reduce the number of items to 103 and to modify the way in which some of the items were phrased. Specifically, items that were very specific to autism were eliminated, since the instrument was intended to be applicable to other neurodevelopmental disorders [e.g., ADHD, ASD, language-specific disorders (LSD), dyspraxia]. After the second round of consultations, the experts reduced the number of items to 94 self-administered items, which were then used to create the initial EPYFEI questionnaire that would be used to start the evaluation process. It took 15 min to fill in the questionnaire.

### Study Population

The 94 items of the initial EPYFEI questionnaire were administered to the parents of 1,732 children. Of these, 1,349 were healthy or “typical” children and 383 were children diagnosed with a neurodevelopmental disorder according to the criteria of the DSM-IV-TR. Such disorders, included ADHD, ASD, LSD, developmental delay, and other neurodevelopmental disorders.

The children were recruited using non-probability convenience sampling. The sample of typical children was obtained from various Spanish preschool and primary education centers by means of meetings with the headmasters of these centers. The sample of children with neurodevelopmental disorders was obtained from several Spanish child therapy centers *via* the occupational therapists and several Spanish associations of parents of children with ADHD, ASD, and LSD. All parents and headmasters received a letter stating the objectives of the study. The parents who agreed to the participation of their children in the study gave their written consent and completed the EPYFEI. The fieldwork took place in the period between April 2015 and December 2017.

### Data Collection

In addition to the 94 items of the EPYFEI, we also obtained children’s sociodemographic and clinical data (i.e., sex, age, educational level) and data on parents’ age and occupation. The children’s clinical diagnoses were obtained from their clinical histories and from data provided by their parents.

### Development of the Final Questionnaire and Internal Consistency

An exploratory factor analysis was conducted to identify the components of sensory processing and executive functions that could discriminate whether the neurodevelopment was typical or not, determining the items that should be retained, and reducing their number as far as possible. For this purpose, we estimated the Kaiser-Meyer-Olkin statistical sample fit (acceptable with values of above 0.5) and conducted the Bartlett sphericity test. The structure was evaluated using an exploratory factor analysis with varimax rotation, extracting the principal components and applying the Kaiser rule (eigenvalues >1) to determine the number of factors. Items were eliminated if they had a factor loading of <0.40 on their own factor. The response options of each item were based on an ordinary five-point Likert scale (0 = never, 1 = almost never, 2 = sometimes, 3 = almost always, 4 = always).

To determine internal consistency, Cronbach’s alpha coefficient was calculated for each of the principal components and the final questionnaire obtained from the factor analysis. A Cronbach’s alpha of 0.70–0.95 is generally accepted as a measure of good internal consistency.

### Construct Validity

Construct validity refers to the degree to which the scoring of the questionnaire is correlated with the measures of other questionnaires in a manner that is consistent with the theoretical hypotheses derived from the concepts being measured. The EPYFEI questionnaire was correlated with the Spanish versions of the behavior assessment system for children (BASC questionnaire for parents, P1 or P2 in parents with children aged 3–6 or 6–12 years old, respectively) (39), the childhood executive functioning inventory (CHEXI) (40, 41), and the short sensory profile 2 (short SP2) (42).

The BASC was designed to facilitate differential diagnosis, to analyze the need to adapt the curriculum to children’s and adults’ emotional and behavioral disorders, and also to support the design of intervention and treatment plans. The BASC questionnaire for parents’ measures the child’s behavior observed at home, thus contributing with a detailed measurement of both adaptive and problem behavior in the family and community spheres. It consists of nine clinical scales (i.e., aggressiveness, hyperactivity, behavior problems, attention problems, atypicality, depression, anxiety, isolation, and somatization, where a higher score represents higher negative or undesirable characteristics) and three adaptive scales (i.e., adaptability, social skills, and leadership, where a higher score means a more positive behavior). The individual use of the components of the BASC is very reliable and psychometrically valid. The reliability of the set of scales in the parents’ questionnaire ranged between 0.70 and 0.80. The test–retest carried out with the Spanish population proved to be highly reliable and indicated good convergent validity. The CHEXI is a questionnaire consisting of 24 items that evaluates four basic aspects of executive functions: working memory, planning, regulation, and inhibitory control. Studies have shown that this instrument has a good internal consistency and factor structure and a good ability to predict academic achievement (40, 43). The CHEXI can be used by both parents and teachers, although in this study only evaluations obtained from parents were used. The internal consistency of the CHEXI for parents and teachers was excellent, with Cronbach’ alpha values of 0.94 and 0.98, respectively (44).

The short SP2 is a standardized instrument used to evaluate a child’s sensory processing patterns in the context of everyday life (42). The information obtained makes it possible to determine how sensory processing may favor or hinder the child’s participation in everyday activities. It is composed of three questionnaires that obtain the opinions and evaluations of parents or carers and teachers, who best know the child’s responses to sensorial interactions that occur throughout the day. The Spanish version of the SP2 can be used with children aged between 3 and 14 years. The short SP2 version, which contains 34 items, has a coefficient of reliability of 0.90 for the Spanish population, and has obtained good results as regards to the test–retest stability coefficients for each of the quadrants in the Short SP2 (search: 0.97; avoidance: 0.96; sensitivity: 0.96; and bystander: 0.93) and for the sensory processing (0.97) and behavior (0.97) subscales (42).

The hypothesis on which this study was based is that the EPYFEI would correlate most strongly with the sensory processing scale of the short SP2 and least strongly with the behavior scale of the short SP2. We also hypothesized that the EPYFEI would show a high correlation with the working memory and inhibition scales of the CHEXI and a lower correlation with the BASC. For this purpose, we calculated Spearman correlation coefficients (*r*). A value of *r* > 0.7 is considered to be good (45).

### Test–Retest Reliability (Repeatability)

Repeatability is the variation in measurements obtained from a single person or instrument in the same item, under the same conditions, and in a short period of time. To determine repeatability, we calculated the interclass correlation coefficient (ICC) with a 95% confidence interval between the scores obtained for the test and the retest in order to assess the stability of the scores over time. A score of ICC > 0.7 was considered acceptable (45).

The test–retest reliability assessment was carried out between July and December 2017 by the parents of 109 children (71 typical and 38 with a neurodevelopmental disorder) from several Spanish child therapy and preschool education centers, using the same procedure mentioned above. The time interval between test and retest was always 2 weeks. These centers were given the final version of the EPYFEI. In addition, the BASC (39), CHEXI (40, 41), and short SP2 questionnaires (3, 42) were administered.

### Floor and Ceiling Effects

The ceiling and floor effects refer to the percentage of individuals who obtained the highest or the lowest possible scores on a test, respectively. We calculated the percentage of children with the highest and lowest possible scores for the total EPYFEI questionnaire and for each of its five factors. If more than 15% of those surveyed had the maximum or minimum scores, the ceiling and floor effects were considered to be present. These effects reduce reliability, since participants with extreme scores cannot be distinguished from each other (46).

### Interpretability

The Mann–Whitney *U* test was used to compare the scores of typical children (i.e., without disorders) and those of children with neurodevelopmental disorders for the total EPYFEI questionnaire and for each of its five factors. We also calculated the ROC curve of the total score in order to determine the ability to predict whether a child had a neurodevelopmental disorder or typical development. We additionally conducted an analysis to determine the best cut-off point to consider that the screening was positive and identified a potential neurodevelopmental disorder.

### Statistical Analysis

Statistical analyses were conducted using the IBM SPSS Statistics (version 23.0, IBM Corp., Armonk, NY, USA). The level of statistical significance was set at *p* < 0.05 (bilateral). Participants’ characteristics were analyzed using simple descriptive statistics.

## Results

### Description of the Sample

Table 1 provides a description of the sample of 1,732 children. Of these, 77.90% (*n* = 1,349) were children with typical development and without any pathology; 51% (*n* = 884) were male. Mean age was 6.6 (SD = 3.39) years. A total of 18.6% (*n* = 323) of the children were preterm.

**Table 1:** Description of the sample (*n* = 1,732).

		Mean	SD
Age (years old)		6.66	2.39
		*n*	%
Group	Typical	1,349	77.9
	Attention deficit hyperactivity disorder	95	5.5
	ASD	84	4.8
	Developmental delay	15	0.9
	Other neurodevelopmental disorders	83	4.8
	Language-specific disorders	106	6.1

Sex	Female	848	49.0
	Male	884	51.0

Age of child (years old)	≤4	397	22.9
	5–7	658	38.0
	≥8	677	39.1

Educational level	Preschool	705	40.7
	Primary	1,027	59.3

Preterm	Yes	323	18.6
	No	1,409	81.4

### Factor Analysis and Internal Consistency

Table 2 shows the results of the factor analysis of the sample of 1,732 children, the five factors identified, the loading factor of each item, the percentage of answers missing, the eigenvalues, and Cronbach’s alpha of the factors and the explained variance after rotation. We identified five factors, which were labeled as follows: (1) Executive attention, working memory, and initiation of actions; (2) sensory processing; (3) emotional and behavioral self-regulation; (4) supervision and problem solving; and (5) inhibitory control. All the items in each factor had a rotated factor loading greater than 0.4. All the factors had an eigenvalue above 1 and a Cronbach’s alpha between 0.74 and 0.94. The total percentage of explained variance after rotation was 56.55%. The percentage missing in the items never exceeded 0.25%.

**Table 2:** Results of the factor analysis.

	Factor loading	Statistics
**Factor 1. Executive attention, working memory, and initiation of actions**		
Has difficulties in understanding the instructions he/she is given to perform tasks	0.691	Eigenvalue: 7.14Cronbach’s alpha: 0.94395% CI (0.939–0.947)Explained variance after rotation %: 20.99
Is not prone to initiate activities; needs to be stimulated or asked to do so	0.656	
Has difficulties following a conversation, activity, or instructions	0.756	
Has difficulties maintaining attention to perform an activity and needs to take breaks or interrupt the activity	0.731	
Has problems in selecting the essential information or necessary objects to perform a task or solve a problem	0.765	
Has difficulties doing things that require a mental effort	0.795	
Has difficulties performing activities that involve several steps	0.664	
Has difficulties remembering information while he/she is performing another activity	0.771	
Takes time to finish activities and needs more time than other children his/her age	0.800	
Needs continuous efforts to perform and finish activities	0.716	
Has difficulties in describing something that happened so that others can understand it easily	0.693	

**Factor 2. General sensory processing**		
Has problems recognizing objects visually	0.649	Eigenvalue: 3.88Cronbach’s alpha: 0.83395% CI (0.821–0.845)Explained variance after rotation %: 11.41
Does not like water at all	0.657	
Touches or rubs the body part, where he/she was touched by somebody	0.613	
Usually leans on himself/herself or on an object or wall to support his/her head, body…. etc	0.691	
Has problems climbing steps, moving, staggers, or has difficulties riding down a slide in the park or elsewhere	0.726	
Struggles to keep his/her balance on uneven ground	0.654	
Injures or hurts himself/herself	0.673	

**Factor 3. Emotional and behavioral self-regulation**		
Protests when does not get own way	0.603	Eigenvalue: 2.97Cronbach’s alpha: 0.818IC 95% (0.804–0.831)Explained varianceafter rotation %: 8.73
Reacts inappropriately to criticism	0.713	
Has rapid mood swings	0.697	
Cries and/or gets frustrated easily	0.703	
Has tantrums	0.703	

**Factor 4. Supervision, correction of actions, and problem solving**		
Cooperates in the performance of activities	0.685	Eigenvalue: 2.77Cronbach’s alpha: 0.74795% CI (0.728–0.765)Explained varianceafter rotation %: 8.14
Revises and corrects activities	0.647	
Knows how to organize his/her free time and play by himself/herself	0.611	
Solve the problems that come up in the activities	0.652	
Performs daily activities, such as getting dressed, washing, eating, etc., without help	0.578	
Recognizes the feelings and needs of others	0.585	

**Factor 5. Inhibitory control**		
Usually hums or makes noises while he/she performs tasks and should be silent	0.556	Eigenvalue: 2.47Cronbach’s alpha: 0.73995% CI (0.719–0.758)Explained varianceafter rotation %: 7.26
Seeks activities involving jumping, crawling, pressing, pushing, or pulling	0.712	
Has difficulties staying still	0.702	
Acts without planning what he/she has to do, in an impulsive way	0.581	
Switches to another activity without finishing the one he/she was performing	0.487	

*95% CI, 95% confidence interval for Cronbach’s alpha. Total explained variance after rotation %, 56.55. All the items have less than 0.5 of missing values*.

### Construct Validity

Table 3 shows the correlation values between the scores of the EPYFEI questionnaire (both the total score and those of its four factors) and the scores obtained for the BASC, CHEXI, and SP2 questionnaires.

**Table 3:** Correlation between the EPYFEI and the BASC, the CHEXI, and the SP2 questionnaires.

	Total score EPYFEI	FACTOR 1	FACTOR 2	FACTOR 3	FACTOR 4	FACTOR 5
	*r*_s_	*r*_s_	*r*_s_	*r*_s_	*r*_s_	*r*_s_
BASC aggressiveness	0.20	0.14	0.13	0.32	−0.19	0.23
BASC hyperactivity	0.46	0.44	0.12	0.41	−0.30	0.56
BASC behavior PROBLEMS	−0.19	−0.08	−0.05	−0.02	−0.50	0.12
BASC attention problems	0.52	0.64	0.15	0.34	−0.28	0.41
BASC atypical	0.65	0.59	0.20	0.0.58	−0.04	0.48
BASC depression	0.41	0.41	0.18	0.44	−0.21	0.29
BASC anxiety	0.39	0.30	0.10	0.34	0.14	0.05
BASC withdrawal	0.12	0.19	0.04	0.10	−0.01	−0.02
BASC Somatization	−0.36	−0.23	−0.01	−0.26	−0.21	−0.21
BASC adaptability	−0.03	−0.08	−0.17	−0.10	0.31	−0.03
BASC social skills	−0.30	−0.36	−0.20	−0.24	0.33	−0.33
BASC leadership	−0.30	−0.39	−0.31	−0.18	0.34	−0.15
BASC exteriorizing problems	0.24	0.24	0.12	0.32	−0.39	0.40
BASC interiorizing problems	0.29	0.31	0.19	0.27	−0.19	0.15
BASC adaptive skills	−0.19	−0.24	−0.27	−0.19	0.36	−0.16
Planning CHEXI	0.42	0.0.59	0.29	0.17	−0.43	0.40
Working memory CHEXI	0.29	0.46	0.29	0.08	−0.51	0.33
Regulation CHEXI	0.65	0.71	0.24	0.48	−0.37	0.67
Inhibition CHEXI	0.58	0.59	0.24	0.45	−0.41	0.64
SP2 seeker	0.65	0.57	0.27	0.41	−0.07	0.68
SP2 avoider	0.59	0.52	0.20	0.48	−0.06	0.49
SP2 sensitive	0.60	0.65	0.35	0.21	−0.11	0.36
SP2 bystander	0.51	0.39	0.27	0.35	−0.02	0.43
SP2 sensorial	0.68	0.0.66	0.60	0.22	−0.74	0.63
SP2 behavioral	0.80	0.85	0.22	0.30	−0.70	0.58

*Spearman’s (rs) correlation; EPYFEI, assessment of sensory processing and executive functions in childhood; BASC, behavior assessment system for children questionnaire for parents; CHEXI, childhood executive functioning inventory; Short SP2, short sensory profile 2; FACTOR 1, executive attention, working memory and initiation of actions; FACTOR 2, general sensory processing; FACTOR 3, emotional and behavioral self-regulation; FACTOR 4, supervision, correction of actions and problem solving; FACTOR 5, inhibitory control*.

Results show that the total score obtained for the EPYFEI has a positive and high correlation with the short SP2 behavioral subscale (0.80, *p* < 0.001), the short SP2 sensory subscale (0.68, *p* = 0.008), the CHEXI regulation subscale (0.65, *p* < 0.001), the BASC atypicality subscale (0.65, *p* < 0.001), the CHEXI inhibition scale (0.58, *p* < 0.001), the BASC attention problems subscale (0.52, *p* < 0.001), and the short SP2 seeker profile (0.65, *p* < 0.001).

The EPYFEI executive attention, working memory, and initiation of actions factor additionally had a positive and high correlation with the short SP2 behavioral subscale (0.85, *p* < 0.001), the short SP2 sensory subscale (0.66, *p* < 0.001), the CHEXI regulation subscale (0.71 p < 0.001), the CHEXI inhibition and planning subscales (0.59, *p* < 0.001), the BASC attention problems subscale (0.64, *p* < 0.001), the BASC atypicality subscale (0.59, *p* < 0.001), and the three short SP2 sensory modulation profiles: seeker pattern (0.57, *p* < 0.001), sensitive pattern (0.65, *p* < 0.001), and avoider pattern (0.52, *p* < 0.001). By contrast, the EPYFEI sensory processing factor had a positive and high correlation with the short SP2 sensory subscale (0.60, *p* = 0.019).

The EPYFEI self-regulation factor showed a positive and moderate correlation with the BASC atypicality subscale (0.58, *p* < 0.001), the CHEXI regulation subscale (0.48, *p* < 0.001), and the short SP2 avoider patterns (0.48, *p* < 0.001). The only factor of the EPYFEI that was negatively correlated with the other scales was supervision and problem solving. It was correlated with the following subscales: BASC behavior (−0.50, *p* < 0.001), CHEXI planning (−0.43, *p* < 0.001), CHEXI working memory (−0.51, *p* < 0.001), CHEXI inhibition (−0.41, *p* < 0.001), short SP2 behavioral (−0.70, *p* < 0.001), and short SP2 sensory (−0.74, *p* < 0.001).

Finally, the EPYFEI lack of inhibitory control factor showed a positive and moderate correlation with the BASC hyperactivity subscale (0.56, *p* < 0.001) and the CHEXI regulation (0.67, *p* < 0.001) and inhibition (0.64, *p* < 0.001) subscales, the short SP2 seeker pattern (0.68, *p* < 0.001), the short SP2 behavioral subscale (0.58, *p* < 0.001), and the short SP2 sensory subscale (0.63, *p* < 0.001).

### Test–Retest Reliability (Repeatability)

Table 4 shows the test–retest differences for the total score and that of the five factors of the EPYFEI questionnaire along with the ICC. All the differences were weak (less than 0.5 points) and none were statistically significant. The ICCs were between 0.75 and 0.93 for the total score and the five factors.

**Table 4:** Average test and retest scores, difference, and ICC.

		Test		Retest		Difference		95% CI diff				95%CI ICC	
	*n*	Mean	SD	Mean	SD	Mean	SD	Ll	Ul	*p*	ICC	Ll	Ul
Total Score	65	33.88	14.71	33.34	14.49	0.54	8.14	−1.46	2.53	0.6	0.916	0.862	0.949
FACTOR 1	66	9.17	9.45	8.91	9.06	0.26	4.69	−0.88	1.40	0.66	0.931	0.888	0.958
FACTOR 2	68	1.91	2.14	1.99	2.07	−0.07	1.79	−0.50	0.36	0.74	0.78	0.643	0.864
FACTOR 3	67	5.88	4.27	5.57	4.20	0.31	3.78	−0.60	1.23	0.5	0.752	0.596	0.847
FACTOR 4	67	12.55	5.22	12.64	5.07	−0.09	3.13	−0.85	0.67	0.82	0.898	0.834	0.937
FACTOR 5	68	4.93	4.13	4.57	4.13	0.35	2.11	−0.15	0.86	0.17	0.93	0.886	0.957

*95% CI diff, 95% confidence interval of the difference; Ll, lower limit; Ul, upper limit; ICC, interclass correlation coefficient; 95% CI ICC, 95% confidence interval of the ICC; FACTOR 1, executive attention, working memory and initiation of actions; FACTOR 2, general sensory processing; FACTOR 3, emotional and behavioral self-regulation; FACTOR 4, supervision, correction of actions and problem solving; FACTOR 5, inhibitory control*.

### Floor and Ceiling Effects

Table 5 shows the maximum and minimum scores obtained for the EPYFEI questionnaire and its five factors, along with the percentage of subjects with the maximum and minimum score. All percentages were lower than 11%, with the exception of the percentage of participants with minimum scores on the sensory processing factor (35%).

**Table 5:** Floor and ceiling effect: percentage of minimum and maximum values.

	*n*	Mean	SD	Median	Min	Max	N of min	N of max	% of min	% of max
Total score	1,707	43.07	18.32	38	6	136	2	4	0.12	0.23
FACTOR 1 score	1,722	10.21	10.75	6	0	46	189	4	10.98	0.23
FACTOR 2 score	1,725	2.71	4.54	1	0	31	615	4	35.65	0.23
FACTOR 3 score	1,720	7.57	4.80	7	0	23	33	4	1.92	0.23
FACTOR 4 score	1,722	15.25	5.21	16	0	29	6	7	0.35	0.41
FACTOR 5 score	1,728	7.42	4.66	7	0	22	70	4	4.05	0.23

*FACTOR 1, executive attention, working memory and initiation of actions; FACTOR 2, general sensory processing; FACTOR 3, emotional and behavioral self-regulation; FACTOR 4, supervision, correction of actions, and problem solving; FACTOR 5, inhibitory control. N of min, number of minimum values; N of max, number of maximum values; % of min, percentage of minimum values; % of max, percentage of maximum values*.

### Interpretability

The mean of the total scores of the EPYFEI questionnaire and its five factors for typical children and those with neurodevelopmental disorders are shown on Table 6. Scores were considerably higher in children with neurodevelopment disorders, with Cohen’s D showing highly different values for all factors (>0.75), with the exception of Factor 4, related to strength components (47).

**Table 6:** Average scores of healthy children and children with a disorder.

	Typical			Disorder			Difference	95% CI diff			
	*n*	Mean	SD	*n*	Mean	SD	Mean	Ll	Ul	*D* Cohen	*p*
Total score	1,336	37.85	13.07	371	61.89	21.86	24.04	22.27	25.82	1.60	<0.001
Score FACTOR 1	1,342	6.58	6.84	380	23.03	12.17	16.44	15.17	17.73	2.05	<0.001
Score FACTOR 2	1,345	1.72	2.62	380	6.19	7.34	4.47	3.72	5.22	1.22	<0.001
Score FACTOR 3	1,343	6.81	4.35	377	10.29	5.33	3.48	2.89	4.06	0.76	<0.001
Score FACTOR 4	1,342	16.08	4.69	380	12.34	5.87	−3.74	−4.38	−3.10	−0.75	<0.001
Score FACTOR 5	1,347	6.67	4.30	381	10.06	4.92	3.39	2.85	3.94	0.76	<0.001

*95% CI diff, 95% confidence interval of the difference; Ll, lower limit; Ul, upper limit; FACTOR 1, executive attention, working memory and initiation of actions; FACTOR 2, general sensory processing; FACTOR 3, emotional and behavioral self-regulation; FACTOR 4, supervision, correction of actions and problem solving; FACTOR 5, inhibitory control*.

**Figure 1:**
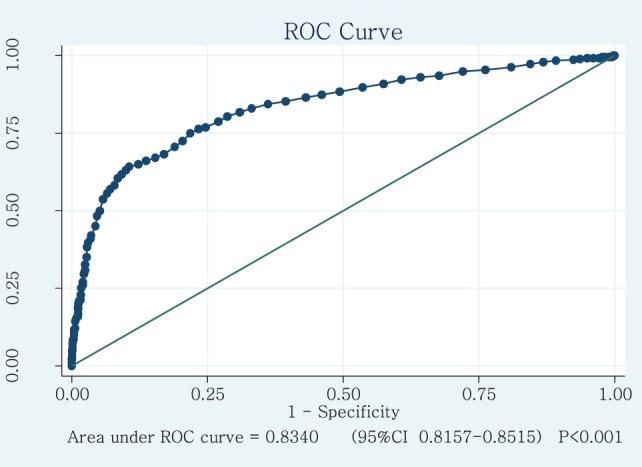
ROC curve with which to determine the predictive value of the assessment of sensory processing and executive functions in childhood scale in the diagnosis of children with a disorder.

In addition, the ROC curve method was used to further explore the discriminant validity of EPYFEI. Typically, an area under the ROC curve equal to 1 corresponds to an instrument that discriminates perfectly between patients and controls, whereas a value of 0.50 indicates that the instrument discriminates with an accuracy no better than chance. An area under the ROC curve >0.70 indicates adequate discrimination. Figure 1 shows the ROC curve used to determine the predictive value of the EPYFEI scale for the diagnosis of children with a neurodevelopmental disorder. The area under the curve was >0.8. Table 7 depicts the calculation of the cut-off points of the total score for the EPYFEI scale according to the different levels of sensitivity and specificity. The optimum cut-off point, which leads to a maximum Youden index, was 46.5 points. The Spanish version of the EPYFEI can be found in Table S1 in Supplementary Material.

**Table 7:** Cut-off points of the assessment of sensory processing and executive functions in childhood questionnaire.

Disorder is ≥	Sensitivity %	Specificity %	Youden I	TP	FP	TN	FN	PPV	NPV
7.5	1.000	0.001	0.001	244	0	2	1,258	100.0	0.1
16.5	0.995	0.016	0.011	243	1	21	1,239	99.5	1.6
29.5	0.949	0.280	0.229	232	12	353	907	94.9	28.0
**46.5**	**0.749**	**0.781**	**0.531**	**183**	**61**	**985**	**275**	**74.9**	**78.1**
55.5	0.617	0.908	0.525	151	93	1,144	116	61.7	90.8
63.5	0.450	0.957	0.407	110	134	1,205	55	45.0	95.7
70.5	0.307	0.975	0.283	75	169	1,229	31	30.7	97.5
77.5	0.210	0.987	0.197	51	193	1,243	17	21.0	98.7
90.5	0.086	0.996	0.083	21	223	1,255	5	8.6	99.6
112.0	0.022	0.999	0.021	5	239	1,259	1	2.2	99.9

*No. with disorder, 371; No. typical, 1,336; Total no., 1,707*.

*Youden I, Youden index; TP, true positives; FP, false positives; TN, true negatives; FN, false negatives; PPV, positive predictive value; NPV, negative predictive value; in bold type, data corresponding to maximum Youden index*.

## Discussion

In this study, we explored the psychometric properties of the EPYFEI, a new instrument to assess sensory processing and executive functions in children aged between 3 and 11 years. After analyzing its items, 34 of the original 108 items were retained in the final version. Results indicate that this questionnaire has good psychometric properties in terms of validity, reliability, and discriminant value for children with typical development and children with neurodevelopmental disorders. Furthermore, the design allowed the development of cut-off scores for the EPYFEI.

Neurodevelopmental disorders are alterations that interfere with the proper maturation and functioning of individuals. They occur from birth and early childhood and do not always show structural lesions. Currently, the DSM-5 has adopted the use of this term to replace the previous term, which referred to disorders with onset in childhood or adolescence. Such disorders are characterized by deficits in development and lead to limitations in specific areas or global limitations for personal, social, academic, or occupational functioning. The interest of the EPYFEI is relevant for the early care of individuals with neurodevelopmental disorders, whose prevalence is considered to be 4.5% in individuals aged 0–5 years and to be as high as 18.5% in individuals aged 6–10 years. The most common pathologies are ADHD (5.36%) and language disorders (3.42%) (48). At present, there are still many children who reach school age with a developmental disorder that has not been previously diagnosed and consequently without benefiting from early care services and programs. In addition, this questionnaire was designed to be used in primary care pediatric services, and can be easily included in monitoring programs for healthy children.

At present, the SP2 is the only standardized instrument validated and adapted to the Spanish population that can be used to assess the sensory processing of children aged between 3 and 14 years (42). Interestingly, conventional tests used to assess executive functions have been criticized for their lack of ecological validity (11). Most of them have focused essentially on experimental paradigms, requiring relatively easy responses to simple tasks, far from the demands of activities of daily living. These types of tests do not make it possible to discern which is the real functional repercussion of the executive functions or deficits that a person may have (11). With this regard, the EPYFEI questionnaire, which assesses sensory processing, was also aimed at evaluating executive functions of children in their daily activities in their context. It focuses on a family perspective, demonstrating that parents are able to report on their children’s skills compared to other children of the same age.

### Reliability and Validity

The five subscales or factors demonstrated have good internal consistency and reliability. They obtained good psychometric values for the individual factors (Attention, Working Memory and Initiation of Actions, Emotional and Behavioral Self-Regulation, Sensory Processing, Supervision, Correction of Actions and Problem Solving, and Inhibitory control) and for the total score of the questionnaire. As regards the subscales, some sensory domains proved to be more reliable than others. The lowest alpha scores were obtained for inhibitory control (0.71–0.75).

### Discriminant Validity

This study provides preliminary evidence of the discriminant validity of the EPYFEI. Validity was demonstrated by the fact that the scores of children with neurodevelopmental disorders significantly differed from those of children with typical development. The total score obtained for the EPYFEI makes it possible to consistently differentiate children with typical development from those with neurodevelopmental disorders, with the cut-off point having been established at 46.5.

### Construct Validity

Overall, the EPYFEI and each of its subscales showed good construct validity. The factor analysis confirmed two basic executive functions: (1) “cold” executive functions: executive attention-working memory, supervision, and problem solving, and inhibitory control and (2) “hot” executive functions: emotional and behavioral self-regulation. It also established the importance of proprioception, tactile, and vestibular processing for the performance of and participation in daily activities of children aged between 3 and 11 years.

### Description of the EPYFEI

The final scale is composed of 34 items, which are grouped into five factors or dimensions: (1) Attention, working memory, and initiation of actions, which includes 11 items; (2) global sensory processing, particularly tactile, proprioceptive, and vestibular information, with seven items; (3) emotional and behavioral self-regulation, which includes five items; (4) organization, execution, supervision, and problem solving in activities of daily living, with six items; and (5) inhibitory control, with five items. Interestingly, the EPYFEI has the same number of items as the short SP2. The short SP2 is specifically divided into two dimensions. One dimension is sensory processing, with 14 items and the other one is related to behavioral responses associated with sensory processing and contains 20 items. Unfortunately, we were unable to find the results of any exploratory or confirmatory factor analyzes of the Spanish version of the short SP2 that may have explained the construct validity of this instrument. A confirmatory factor analysis of the English version of the SP2 concluded that this instrument adjusts well to the four dimensions of the sensory processing model: seeker, avoider, sensitive, and low register (49). An exploratory factor analysis of a previous version of this instrument, the sensory profile (SP), revealed a 9-factor solution. The factors were (1) Sensory searching, which explained 22.2% of the variance; (2) being emotionally reactive, which explained 6.1% of the variance; (3) hypotonia and reduced resistance, which explained 4% of the variance; (4) oral sensitivity, which explained 3.3% of the variance; (5) inattention, which explained 2.7%; (6) low register, which explained 2.6%; (7) sensory sensitivity, which explained 2.5%; (8) sedentary behavior, which explained 2.3%, and (9) perception and fine motor skills, which explained 2.1% (50). When compared to the short SP2, there is a similarity between the items found in several factors. The first factor of the SP, called “sensory searching,” reflects some behaviors with a lack of inhibitory control that could also be conceptualized as executive functions. Some examples are excessive movement and interference with daily activities, the performance of excessively risky activities while playing, the continual search for activities involving movement, finding enjoyment in making noises, and always touching things or other people. The second factor indicated by Dunn and Brown as being emotionally reactive could be equivalent to our third factor, called “emotional and behavioral self-regulation or self-control” (e.g., poor tolerance of frustration, oversensitivity to criticism, etc.). However, we found only one specific factor related to sensory processing. This factor included only one item related to visual processing, two items related to vestibular processing, and four items related to tactile and proprioceptive processing in the EPYFEI. Moreover, the correlation analysis showed a positive and high correlation between the total score obtained for the EPYFEI and all its factors and the total score for the short SP2. We also observed a strong and positive correlation between the sensory and behavioral scale of the short SP2 and the executive attention-working memory, supervision and problem solving, and inhibitory control subscales of the EPYFEI. This correlation was not found with the behavioral and emotional self-regulation factor.

There is no reduced version of the parent-reported sensory processing measure-preschool (SPM-P) and sensory processing measure (SPM) (51). This instrument has not been adapted to the Spanish culture and no evaluation studies have been carried out with the Spanish population. Nevertheless, a similar exploratory factor analysis to that presented for the EPYFEI was conducted with the American version. The SPM-P includes 75 items grouped into eight dimensions: social participation, sight, hearing, touch, taste and smell, proprioception, vestibular, planning, and ideation. The exploratory factor analysis of the American version of the SPM-P showed 8 factors, of which the first explained 20% of the variance, labeled as “problem behaviour;” the second referred to social participation, the third to proprioception, and the fourth to planning and ideation. The items regarding hearing, vestibular functions (i.e., balance and movement), and sight had less explanatory weight. Moreover, there is no factor related to touch, since these items are distributed among other factors, as also occurs with the items regarding taste and smell (52). The SPM also has 75 items, which are grouped into the same eight dimensions as in the SPM-P. The factor analysis of this instrument identified seven factors. The main one refers to problem behavior and explained 27.7% of the total variance. The second factor represents proprioception, the third is social participation, and the fourth contains items concerning planning and the ideation of plans of action. The items with the least explanatory power are those that refer to audio, visual, and tactile processing. The authors of the questionnaire reached no conclusions as to what the fifth factor may refer to. None of the factors appeared to clearly show the items of balance and movement. Once again, similarly to what occurs with the SPM-P, the items related to taste and smell were not contained in any of the factors (51). Both studies have the same factors for preschool and school-aged children. The results mentioned above could be considered similar to those that we obtained in our study, since problem behavior is usually the product of alterations concerning attention and the self-regulation or self-control of behavior.

Furthermore, the factor analysis of the EPYFEI showed three of the factors contained in the CHEXI (41): working memory, regulation, and inhibition. There was also a positive and strong correlation between the executive attention-working memory factor of the EPYFEI (Factor 1) and the working memory, inhibition, and regulation scales of the CHEXI. Both the total score of the EPYFEI and its executive attention and working memory subscale had a positive and strong relationship with the BASC attention problems subscale (39). Moreover, the BASC atypicality subscale was strongly and positively correlated with the total score and the EPYFEI executive attention-working memory and emotional self-regulation subscales. Finally, there was also a high and strong correlation between the results obtained for the inhibition subscale and the total score obtained for the EPYFEI with the hyperactivity subscale. This could be interpreted as support for evidence that the EPYFEI is able to distinguish between neurodevelopmental disorders (e.g., ADHD, ASD, LSD) and developmental delay, which were included in our clinical sample.

### Implications in Practice

The early identification of children who are at risk of having a deficit of executive functions and sensory processing has important clinical implications. First, because these deficits have been associated with the later diagnosis of a neurodevelopmental disorder (18). Second, because such identification would facilitate the commencement of appropriate treatment, thus preventing future consequences in the development of both preschool and school children.

The use of questionnaires may be a first step in the identification process that is relatively quick, low cost, and easy to implement. Moreover, the EPYFEI is useful to obtain information about the daily performance of sensory processing and executive functions. It is a reliable and valid instrument with the potential to assist in the early detection of children with sensory processing and executive function disorders.

An advantage of the use of EPYFEI is that when parents answer the questionnaire they develop a greater awareness of the importance of diversions from typical development in their children. Their responses are very important because they know the most important milestones of their children’s development (48).

Our results showed that executive functions are essential for the performance of everyday activities in children aged between 3 and 11 years. These cognitive processes can explain a greater amount of variability between children with typical development and those with neurodevelopmental disorders than sensory processing does. Additionally, it seems that tactile, proprioceptive, and vestibular processing are better able to screen for neurodevelopmental disorders in that age range than other sensory processing patterns (e.g., visual, hearing, oral). In this regard, the EPYFEI could be useful as a first step such as screening, when there is a clinical need to determine if there is or not a dysfunction in performance. If the screening with EPYFEI is positive, the following steps could include the use of other tools that confirm which neurodevelopmental disorder we are dealing with.

## Limitations and Future Work

Although this study analyzed a clinical sample, further research with several larger groups of children with neurodevelopmental disorders is necessary. It would also be advisable to carry out pre-post treatment studies in order to determine the sensitivity of the EPYFEI as a measure of the results of treatment.

It would also be interesting to carry out future studies with a confirmatory factor analysis of the EPYFEI. We consider that it would be very useful to be able to develop a questionnaire for babies and another one for teachers that will enable the latter to assess the sensory processing and executive functions of children in the school context.

## Conclusion

The EPYFEI makes a unique contribution to the understanding of neurodevelopmental disorders, in which simultaneous difficulties concerning sensory processing and executive functions in activities of daily living occur in children. The instrument provides a means to distinguish children with neurodevelopmental disorders from those with typical development. The psychometric results confirm its internal consistency, test–retest reliability, construct validity, and discriminatory validity according to the information provided by parents. All of this can lead to a potential reduction in health spending and/or investment as well as in the clinical occurrence of mental and physical health problems. In the area of education, the use of this measure could improve inclusive education through a better understanding neurodevelopmental disorders. From a time-related perspective, it may lead to an opportunity to provide an early response to the needs of children and their families using a multidisciplinary approach agreed by consensus, thus avoiding the long process of attempting to find the right treatment. Future studies should aim at providing evidence of its use in other clinical populations and its cross-cultural validity.

## Ethics Statement

The typical sample was obtained by means of meetings with the heads of the education and/or Parents’ centers, who were given a letter stating the objectives of the study together with an informed consent form for each participant. All parents who completed the questionnaire did so voluntarily and gave their written consent to participate in the study. The study was approved within a larger study by the Ethics and Clinical Research Committee of the Hospital Ntra. Sra. del Prado de Talavera de la Reina, Toledo (Spain).

## Author Contributions

All authors have contributed to the different phases of the study, in the design, data collection, analysis, and preparation of the manuscript.

## Conflict of Interest Statement

The authors declare that the research was conducted in the absence of any commercial or financial relationships that could be construed as a potential conflict of interest.

## References

[1] Zingerevich C Greiss-Hess L Lemons-Chitwood K Harris SW Hessl D Cook K Motor abilities of children diagnosed with fragile X syndrome with and without autism. J Intellect Disabil Res (2009) 53(1):11–8. 10.1111/j.1365-2788.2008.01107.x 18771512 PMC2614297

[2] Dunn W Supporting children to participate successfully in everyday life by using sensory processing knowledge. Infants Young Children (2007) 20(2):84–101. 10.1097/01.IYC.0000264477.05076.5d

[3] Dunn W Sensory Profile 2: User’s Manual. USA: Pearson (2014).

[4] Simon SS Merging of the senses. Front Neurosci (2008) 2(1):13–4. 10.3389/neuro.01.019.20088 18982098 PMC2570067

[5] Ahn RR Miller LJ Milberger S McIntosh DN. Prevalence of parents’ perceptions of sensory processing disorders among kindergarten children. Am J Occup Ther (2004) 58(3):287–93. 10.5014/ajot.58.3.287 15202626

[6] Ben-Sasson A Cermak SA Orsmond GI Tager-Flusberg H Kadlec MB Carter AS. Sensory clusters of toddlers with autism spectrum disorders: differences in affective symptoms. J Child Psychol Psychiatry (2008) 49(8):817–25. 10.1111/j.1469-7610.2008.01899.x 18498344

[7] Schoen SA Miller LJ Brett-Green BA Nielsen DM. Physiological and behavioral differences in sensory processing: a comparison of children with autism spectrum disorder and sensory modulation disorder. Front Integr Neurosci (2009) 3:29. 10.3389/neuro.07.029.2009 19915733 PMC2776488

[8] American Occupational Therapy Association. Occupational therapy practice framework: domain and process (3rd ed.). Am J Occup Ther (2014) 68(Suppl 1):S1–S48. 10.5014/ajot.2014.682006

[9] Boyd BA McBee M Holtzclaw T Baranek GT Bodfish JW. Relationships among repetitive behaviors, sensory features, and executive functions in high functioning autism. Res Autism Spectr Disord (2009) 3(4):959–66. 10.1016/j.rasd.2009.05.003 21475640 PMC3071047

[10] Burgess PW Veitch E de Lacy Costello A Shallice T. The cognitive and neuroanatomical correlates of multitasking. Neuropsychologia (2000) 38(6):848–63. 10.1016/S0028-3932(99)00134-7 10689059

[11] Chan RC Shum D Toulopoulou T Chen EY. Assessment of executive functions: review of instruments and identification of critical issues. Arch Clin Neuropsychol (2008) 23(2):201–16. 10.1016/j.acn.2007.08.010 18096360

[12] Posner J A different approach to rising rates of ADHD diagnosis. J Am Acad Child Adolesc Psychiatry (2014) 53(6):697 10.1016/j.jaac.2014.02.006 24839888

[13] Rothbart MK Sheese BE Rueda MR Posner MI. Developing mechanisms of self-regulation in early life. Emot Rev (2011) 3(2):207–13. 10.1177/1754073910387943 21892360 PMC3164871

[14] Bechara A Damasio H Damasio AR Lee GP. Different contributions of the human amygdala and ventromedial prefrontal cortex to decision-making. J Neurosci (1999) 19(13):5473–81. 10377356 10.1523/JNEUROSCI.19-13-05473.1999 PMC6782338

[15] Lezak M Howieson D Bigler E Tranel D Neuropsychological Assessment (Fifth Edition ed.). New York: Oxford University Press (2012).

[16] Goel V Grafman J Tajik J Gana S Danto D. A study of the performance of patients with frontal lobe lesions in a financial planning task. Brain (1997) 120(Pt 10):1805–22. 10.1093/brain/120.10.1805 9365372

[17] Green MF Kern RS Braff DL Mintz J Neurocognitive deficits and functional outcome in schizophrenia: are we measuring the “right stuff?”. Schizophr Bull (2000) 26(1):119–36. 10.1093/oxfordjournals.schbul.a033430 10755673

[18] Johnson MH. Executive function and developmental disorders: the flip side of the coin. Trends Cogn Sci (2012) 16(9):454–7. 10.1016/j.tics.2012.07.001 22835639

[19] Nakagawa A Sukigara M Miyachi T Nakai A. Relations between temperament, sensory processing, and motor coordination in 3-year-old children. Front Psychol (2016) 7:623. 10.3389/fpsyg.2016.00623 27199852 PMC4850168

[20] Baranek GT David FJ Poe MD Stone WL Watson LR. Sensory experiences questionnaire: discriminating sensory features in young children with autism, developmental delays, and typical development. J Child Psychol Psychiatry (2006) 47(6):591–601. 10.1111/j.1469-7610.2005.01546.x 16712636

[21] Adams JN Feldman HM Huffman LC Loe IM. Sensory processing in preterm preschoolers and its association with executive function. Early Hum Dev (2015) 91(3):227–33. 10.1016/j.earlhumdev.2015.01.013 25706317 PMC4392005

[22] Wickremasinghe AC Rogers EE Johnson BC Shen A Barkovich AJ Marco EJ. Children born prematurely have atypical sensory profiles. J Perinatol (2013) 33(8):631–5. 10.1038/jp.2013.12 23412641 PMC3738436

[23] Bolaños C Gomez MM Ramos G Rios Del Rio J. Developmental risk signals as a screening tool for early identification of sensory processing disorders. Occup Ther Int (2016) 23(2):154–64. 10.1002/oti.1420 26644234

[24] Ashburner J Ziviani J Rodger S. Sensory processing and classroom emotional, behavioral, and educational outcomes in children with autism spectrum disorder. Am J Occup Ther (2008) 62(5):564–73. 10.5014/ajot.62.5.564 18826017

[25] Ashburner JK Rodger SA Ziviani JM Hinder EA. Comment on: “An intervention for sensory difficulties in children with autism: a randomized trial” by Schaaf et al. (2013). J Autism Dev Disord (2014) 44(6):1486–8. 10.1007/s10803-014-2083-0 24610043

[26] Perry W Minassian A Lopez B Maron L Lincoln A. Sensorimotor gating deficits in adults with autism. Biol Psychiatry (2007) 61(4):482–6. 10.1016/j.biopsych.2005.09.025 16460695

[27] Miller LJ Nielsen DM Schoen SA. Attention deficit hyperactivity disorder and sensory modulation disorder: a comparison of behavior and physiology. Res Dev Disabil (2012) 33(3):804–18. 10.1016/j.ridd.2011.12.005 22236629

[28] Parush S Sohmer H Steinberg A Kaitz M. Somatosensory function in boys with ADHD and tactile defensiveness. Physiol Behav (2007) 90(4):553–8. 10.1016/j.physbeh.2006.11.004 17198716

[29] Yochman A Alon-Beery O Sribman A Parush S. Differential diagnosis of sensory modulation disorder (SMD) and attention deficit hyperactivity disorder (ADHD): participation, sensation, and attention. Front Hum Neurosci (2013) 7:862. 10.3389/fnhum.2013.00862 24379772 PMC3863757

[30] Shochat T Tzischinsky O Engel-Yeger B. Sensory hypersensitivity as a contributing factor in the relation between sleep and behavioral disorders in normal schoolchildren. Behav Sleep Med (2009) 7(1):53–62. 10.1080/15402000802577777 19116801

[31] Bar-Shalita T Vatine JJ Parush S Deutsch L Seltzer Z. Psychophysical correlates in adults with sensory modulation disorder. Disabil Rehabil (2012) 34(11):943–50. 10.3109/09638288.2011.629711 22149534

[32] Bundy AC Shia S Qi L Miller LJ. How does sensory processing dysfunction affect play? Am J Occup Ther (2007) 61(2):201–8. 10.5014/ajot.61.2.201 17436842

[33] Schoen SA Miller LJ Sullivan JC. Measurement in sensory modulation: the sensory processing scale assessment. Am J Occup Ther (2014) 68(5):522–30. 10.5014/ajot.2014.012377 25184464 PMC4153553

[34] Miller LJ Nielsen DM Schoen SA Brett-Green BA. Perspectives on sensory processing disorder: a call for translational research. Front Integr Neurosci (2009) 3:22. 10.3389/neuro.07.022.2009 19826493 PMC2759332

[35] Koenig KP Rudney SG. Performance challenges for children and adolescents with difficulty processing and integrating sensory information: a systematic review. Am J Occup Ther (2010) 64(3):430–42. 10.5014/ajot.2010.09073 20608274

[36] Reynolds S Lane SJ. Diagnostic validity of sensory over-responsivity: a review of the literature and case reports. J Autism Dev Disord (2008) 38(3):516–29. 10.1007/s10803-007-0418-9 17917804

[37] White BP Mulligan S Merrill K Wright J. An examination of the relationships between motor and process skills and scores on the sensory profile. Am J Occup Ther (2007) 61(2):154–60. 10.5014/ajot.61.2.154 17436837

[38] Terwee CB Bot SD de Boer MR van der Windt DA Knol DL Dekker J Quality criteria were proposed for measurement properties of health status questionnaires. J Clin Epidemiol (2007) 60(1):34–42. 10.1016/j.jclinepi.2006.03.012 17161752

[39] Reynolds C Kamphaus R BASC: Sistema de Evaluación de la Conducta de Niños y Adolescentes [The BASC: A system to assess the behavior of children and adolescents]. Madrid: TEA Ediciones (2005).

[40] Catale C Meulemans T Thorell LB. The childhood executive function inventory: confirmatory factor analyses and cross-cultural clinical validity in a sample of 8- to 11-year-old children. J Atten Disord (2015) 19(6):489–95. 10.1177/1087054712470971 23355496

[41] Thorell LB Nyberg L. The childhood executive functioning inventory (CHEXI): a new rating instrument for parents and teachers. Dev Neuropsychol (2008) 33(4):536–52. 10.1080/87565640802101516 18568903

[42] Dunn W Perfil Sensorial -2. Madrid: Pearson (2016).

[43] Thorell LB Rydell AM Bohlin G. Parent-child attachment and executive functioning in relation to ADHD symptoms in middle childhood. Attach Hum Dev (2012) 14(5):517–32. 10.1080/14616734.2012.706396 22856621

[44] Tonietti-Trevisan B Martins-Dias N Almeida-Berberina A Gotuzo-Seabra A Chilhood executive functioning inventory: Adaptacao e Propiedades psicométricas da Versao Brasileira. Psico-USF, Bragnaca Paulista (2017) 22(1):62–74. 10.1590/1413-82712017220106

[45] García J López J Jiménez F Ramírez Y Lino L Reding A In: México DF, editor. Metodología de la investigación: Bioestadística y bioinformática en ciencias médicas y de la salud (2da. Ed.). Mc Graw Hill Education (2014).

[46] McHorney C Tarlov AR Individual-patient monitoring in clinical practice: Are available health status surveys adequate? Qual Life Res (1995) 4(4):293–307. 10.1007/BF01593882 7550178

[47] Argimón-Pallàs JM Jiménez-Vila J Métodos de investigación clínica y epidemiológica (3ª Edición). Barcelona: Elsevier (2004).

[48] Carballal-Mariño M Gago Ageitos A Ares Alvarez J Del Rio Garma M García Cendón C Goicoechea Castaño A [Prevalence of neurodevelopmental, behavioural and learning disorders in Pediatric Primary Care]. An Pediatr (Barc) (2017). 10.1016/j.anpedi.2017.10.007 29169978

[49] Dunn W Little L Dean E Robertson S Evans B. The state of the science on sensory factors and their impact on daily life for children: a scoping review. OTJR (Thorofare N J) (2016) 36(2 Suppl):3S–26S. 10.1177/1539449215617923 27504990

[50] Dunn W Brown C. Factor analysis on the sensory profile from a national sample of children without disabilities. Am J Occup Ther (1997) 51(7):490–495;discussion496–499. 10.5014/ajot.51.1.25 9242854

[51] Parham LD Ecker C Miller H Henry DA Glennon TJ Sensory Processing Measure. Los Angeles: WPS (2007).

[52] Henry D McClary M The sensroy processing measure-preschool (SPM-P). Part two: test-retest and collective collaborative empowerment. Including a facther’s perspective. J Occup Ther Sch Early Interv (2011) 4(1):53–70. 10.1080/19411243.2011.576891

