# Supplementary material for: Assessment of Sensory Processing and Executive Functions in Childhood: Development, Reliability, and Validity of the EPYFEI
Source: Front Pediatr. 2018 Mar 23;6:71. doi: 10.3389/fped.2018.00071 (PMC5876237; doi:10.3389/fped.2018.00071)
Supplement: Supplementary file 1 [file table_1.docx]

Supplementary Material

Table S1. Original Spanish Items

|  |
| --- |
| **Factor 1. Atención Ejecutiva, Memoria de Trabajo e Inicio de Acciones** |
| Tiene dificultad para entender las instrucciones necesarias para realización de tareas |
| Le cuesta iniciar las actividades, necesita que le estimulen o se lo indiquen |
| Muestra dificultades para seguir el hilo de una conversación |
| Le cuesta mantener la atención para realizar una actividad y necesita hacer descansos o interrupciones en la misma |
| Le cuesta seleccionar la información esencial u objetos necesarios para realizar una tarea o problema |
| Tiene dificultades para hacer actividades en las que necesite concentrarse |
| Tiene dificultades para ejecutar tareas que requieren más de un paso |
| Tiene dificultades para recordar la información mientras está realizando alguna otra actividad |
| Tarda en terminar las actividades. Necesita más tiempo que los niños de su edad |
| Necesita esfuerzos continuos para realizar y terminar las actividades |
| Le resulta difícil explicar a los demás de una manera sencilla un acontecimiento o experiencia vivida |
| **Factor 2. Procesamiento Sensorial General** |
| Le cuesta reconocer visualmente los objetos |
| Rechaza realizar o participar en actividades en las que se exponga al agua (ducharse, ir a la piscina, etc.) |
| Se toca o frota la parte del cuerpo donde le han tocado |
| Habitualmente se apoya en sí mismo o en algún objeto o pared para sostener la cabeza, el cuerpo... etc. |
| Le cuesta subir escalones, moverse, titubea o le cuesta lanzarse por un columpio o un tobogán en el parque u otros espacios |
| Le cuesta mantener el equilibrio en terrenos desnivelados |
| Se agrede y/o o se autolesiona |
| **Factor 3. Auto-regulación Emocional y Conductual** |
| Protesta cuando las cosas no salen como quiere |
| Reacciona inadecuadamente a la crítica |
| Cambia de humor con facilidad |
| Tolera mal la frustración y llora fácilmente |
| Se enfada con frecuencia o se muestra contrariado |
| **Factor 4. Supervisión, Corrección de Acciones y Resolución de Problemas** |
| Coopera en la realización de actividades |
| Revisa y corrige las actividades |
| Sabe organizarse el tiempo libre y jugar por sí mismo |
| Resuelve los problemas que surgen en las actividades |
| Realiza las actividades diarias de vestirse, lavarse, comer. etc. sin ayuda |
| Reconoce los sentimientos y necesidades de los otros |
| **Factor 5. Control Inhibitorio** |
| Habitualmente está tarareando o haciendo ruidos mientras realiza tareas y debería estar en silencio |
| Busca realizar actividades que incluyan saltar, arrastrarse, apretar, empujar o tirar |
| Tiene dificultad para permanecer quieto |
| Actúa de manera impulsiva, sin planificar lo que tiene que hacer |
| Cambia de actividad sin terminar la que estaba realizando |
